# Effect of Multi-Frequency Whole-Body Vibration on Muscle Activation, Metabolic Cost and Regional Tissue Oxygenation

**DOI:** 10.1109/access.2020.3011691

**Published:** 2020-07-24

**Authors:** HIMANSHU SAXENA, KEVIN R. WARD, CHANDRAMOULI KRISHNAN, BOGDAN I. EPUREANU

**Affiliations:** 1 Department of Mechanical Engineering, University of Michigan, Ann Arbor, MI 48109, USA; 2 Michigan Center for Integrative Research in Critical Care, University of Michigan, Ann Arbor, MI 48109, USA; 3 Department of Emergency Medicine, University of Michigan, Ann Arbor, MI 48109, USA; 4 Department of Physical Medicine and Rehabilitation, Michigan Medicine, University of Michigan, Ann Arbor, MI 48109, USA; 5 Department of Biomedical Engineering, University of Michigan, Ann Arbor, MI 48109, USA; 6 Robotics Institute, University of Michigan, Ann Arbor, MI 48109, USA

**Keywords:** Early mobilization, exoskeleton, muscle atrophy, post intensive care syndrome, rehabilitation, tonic vibration reflex

## Abstract

Prolonged immobilization from a critical illness can result in significant muscle atrophy. Whole-body vibration (WBV) could potentially attenuate the issue of muscle atrophy; however, there exists no device that could potentially provide WBV in supine position that is suitable for critically ill patients. Hence, the purpose of this study was to develop a new wearable suit, called therapeutic vibration device (TVD), that can provide WBV in supine position and test its effects on physiologic markers of physical activity including muscle activation, oxygen consumption (VO_2_), and regional hemoglobin oxygen saturation (rSO_2_). The prototype TVD delivered multi-frequency WBV axially to 19 healthy participants in supine position for 10 minutes simultaneously at 25 Hz/4.2 g_rms_ on the feet and 15 Hz/0.7 g_rms_ on the shoulders. Muscle activation was recorded by electromyography (EMG), VO_2_ was measured by indirect calorimetry and rSO_2_ was recorded by near-infrared spectroscopy. Recordings were collected from each participant from multiple body locations, on three separate days, at baseline and during the intervention. Acceleration was also recorded to gain insight into transmissibility and coherence. Repeated-measures ANOVA using Bonferroni correction revealed that the muscle activity significantly increased by 4% - 62% (*p* < 0.05), VO_2_ improved by 22.3% (*p* < 0.05) and rSO_2_ increased by 1.4% - 4.5% (*p* < 0.05) compared to baseline. WBV provided by the TVD is capable of producing physiologic responses consistent with mild physical activity. Such effects could potentially be valuable as an adjunct to physical therapy for early mobilization to prevent atrophy occurring from prolonged immobilization.

## I. INTRODUCTION

During critical illness, patients who are immobilized or incapacitated for prolonged periods develop severe complications including neuromuscular weakness, contractures, and muscle atrophy [1]-[3]. Complete immobilization leads to complications such as loss of muscle strength by 10%-15% each week [1], [3], approximately 40% strength decline within a month [1], [3], and up to a 27% reduction in maximal O_2_ uptake [1]. Despite the reduction in mortality rates [4]-[6], ICU-acquired weakness, functional impairment, and cognitive dysfunction continue to increase [3]-[6]. These sequelae are aspects of the recently defined post-intensive care syndrome (PICS) [4] and may contribute to increased duration of mechanical ventilation, increased length of hospital stay, poor quality of life among survivors, and an increased rate of readmissions [1], [4], [5]. To address PICS, an institutional culture of early mobility and activity is advocated [2], [4], [6] in addition to proactively educating patients and their families [4]-[6]. Early mobilization in the form of walking and cycle ergometry has been demonstrated to significantly reduce polyneuropathy and atrophy associated with PICS, which in turn has demonstrated a reduction in the time patients spend on mechanical ventilation and the overall length of hospital stay [2], [3]. However, these approaches to early mobilization are labor-intensive and cannot be introduced in heavily sedated or unconscious patients.

Whole-body vibration (WBV) may facilitate the rehabilitation process by enhancing muscle activity [7]-[19], increasing metabolic expenditure [20]-[32], improving blood flow and tissue oxygenation [22], [32]-[40], and improving bone mineral density and content [41]-[45]. An increase in muscle activity has been attributed to the tonic vibration reflex [13], [20], [46] or a similar characteristic activity [8], [13], [47]. Evidence suggests that vibrations may produce adequate muscle contraction via spinal reflex loops [13], [15] that may be sufficient to reduce or prevent muscle weakness caused by prolonged immobilization [15], [16]. Thus, WBV may serve as an effective treatment in rehabilitating immobilized patients. Commercially available vibration platforms and pads apply vibrations for alert subjects in a standing or seated position. However, for supine and immobilized patients, these devices have no or very limited body preloading capability and cannot target the upper-extremities. Moreover, the vibration transmissibility to remote parts of the body is attenuated due to the adopted posture and the vibration delivery mechanism. This, in turn, may reduce the effects of vibration on the ability to engage a larger percentage of muscle groups. The use of these existing devices in a clinical environment for immobilized patients poses a significant challenge.

To address these issues, we created a prototype therapeutic vibration device capable of delivering WBV at multiple frequencies simultaneously at distal ends of the body, i.e., feet and shoulders, under axial loading. We also tested the effects of therapeutic vibration device on physiologic markers such as muscle activity, regional hemoglobin oxygen saturation, and whole-body metabolism including oxygen consumption as indicators of physical activity. We hypothesized that low frequency WBV applied axially to large muscle groups in the supine position will increase muscle activation, regional tissue oxygenation, and oxygen consumption.

## II. MATERIALS AND METHODS

### A. PARTICIPANTS

The study was conducted at the University of Michigan, Ann Arbor by the Michigan Center for Integrative Research in Critical Care with approval from the Institutional Review Board of the University of Michigan Medical School (IRBMED). The study was also registered as a clinical trial (clinicaltrials.gov, identifier: NCT03479008). All participants provided written informed consent. The exclusion criteria were: known pregnancy, acute spinal cord injury, acute vertebral body fracture or injury, acute stroke or intracerebral hemorrhage, hemodynamic instability or other events/conditions (e.g., deep vein thrombosis, neurological disorders, and other heart conditions) believed by the care team to warrant exclusion. Out of the 22 participants who were enrolled in the study, 3 participants withdrew due to nausea and discomfort after attending the first session. Data from these participants were excluded from the analysis. 19 medically healthy participants, [9 women and 10 men, age, 51.7 ± 19.5 yr; height, 172.9 ± 8 cm; mass, 76.2 ± 13.9 kg; body mass index (BMI), 25.4 ± 3.6 kg/m^2^ (mean ± SD)] successfully completed the study. An a priori power analysis using G*Power 3.1 with an effect size specification as in SPSS indicated that this sample size yielded a power (*β*) > 80% to detect statistical significance at *α* = 0.05. We used repeated measures (within factors) statistical test for the power analysis with an effect size f(U) = 0.734 and nonsphericity correction *ε* = 1. The effect size f(U) was computed directly in G*Power 3.1 using previously reported effect size (partial *η*^2^ = 0.35) for changes in oxygen consumption due to WBV in active young adults [28].

### B. THERAPEUTIC VIBRATION DEVICE

**FIGURE 1.**
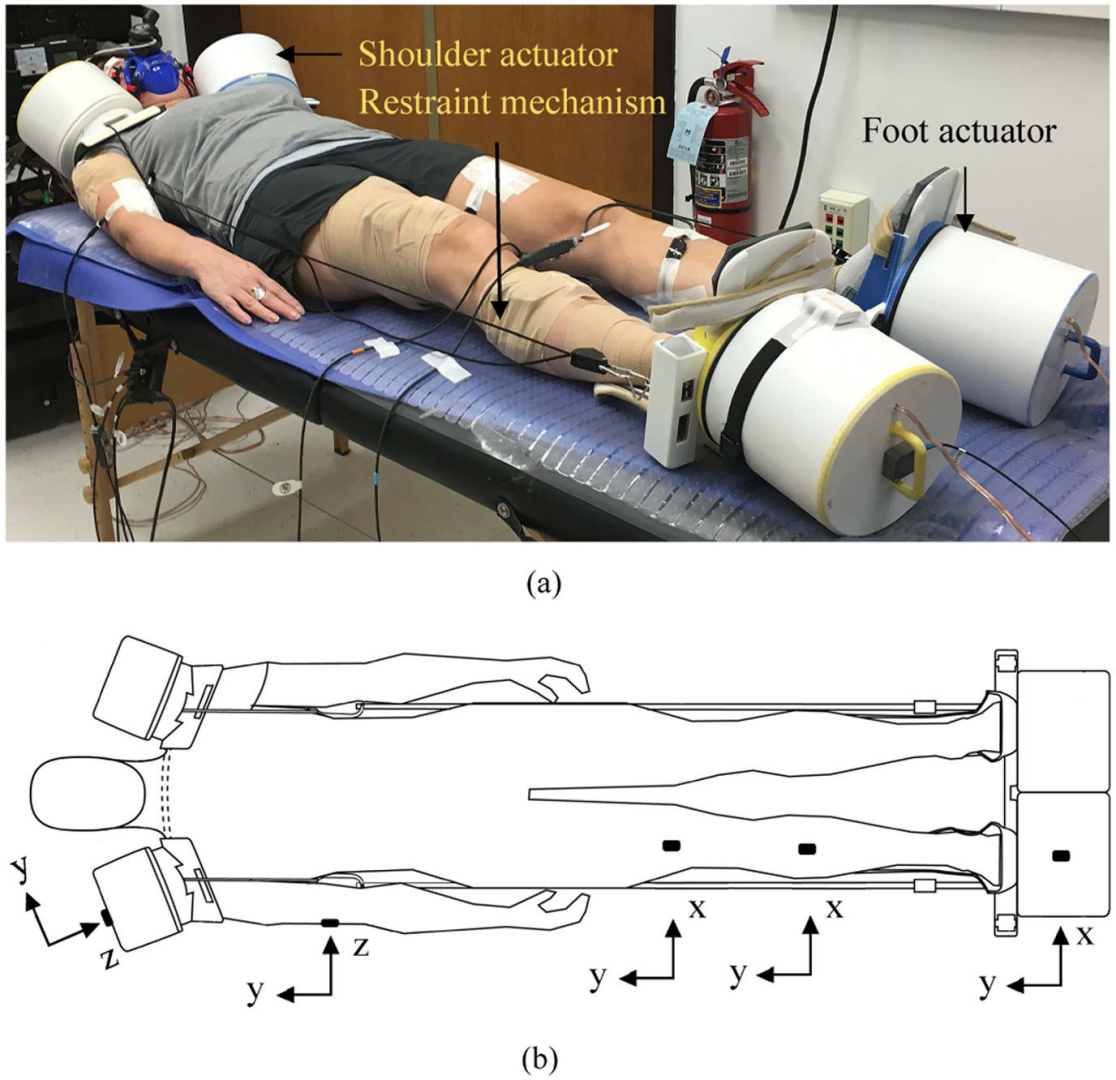
(a) A picture showing the therapeutic vibration device delivering whole-body vibration using inertial actuators to a participant in a supine position. The actuators are preloaded using a restraint mechanism while the participant remains passive. (b) Triaxial accelerometer mounting and coordinate axes orientation with respect to the direction of vibration – i.e., in y-axis on feet and z-axis on shoulders, respectively. They are mounted on foot shaker, tibialis anterior, rectus femoris, brachialis and shoulder shaker.

The device contains four commercially available inertial actuators (ButtKicker Concert and ButtKicker mini Concert, Guitammer Company, USA) housed in custom-designed enclosures. These are assembled with their respective body interfaces, i.e. ankle braces and shoulder cups. A shoulder cup with actuator assembly was mounted on each shoulder. Similarly, an ankle brace with actuator assembly was strapped onto each foot. The device was preloaded using a non-elastic, non-rigid strap restraint mechanism. The inertial actuators were capable of generating vibrations at frequencies between 10 and 100 Hz (Fig. 1). We specifically chose the shoulder and feet combination as it is less obtrusive to bedridden patients. This is also the best configuration for longitudinal transmission of WBV, which has been shown to induce many positive physiologic effects. Actuators at the back, waist, or hips could induce shear forces/stresses that could potentially be detrimental to the patient (e.g., skin breakdown) and hence, those sites were not chosen.

### C. PROTOCOL

**FIGURE 2.**
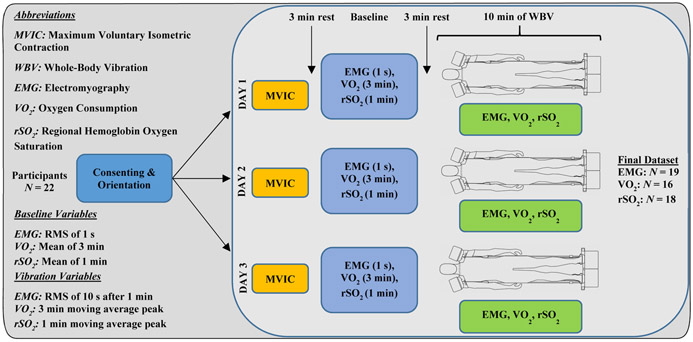
A schematic of the study design.

The schematic of the experimental protocol is provided in Fig. 2. Three sessions were conducted with each participant on three separate days. During the first session, participants were familiarized with the protocol and the device and were trained to carry out maximal voluntary isometric contractions (MVIC) of several muscle groups, in addition to delivering WBV. Participants performed two sets of MVIC tests for 5 seconds per muscle while verbal encouragement was given. Between each protocol activity, a break interval of at least 3 minutes was provided. Participants then laid passively in a relaxed supine position, with their hands on either side of the trunk, without any voluntary contraction for the duration of vibration for 10 minutes (Fig. 1). Based on our pilot experiments, the vibration frequencies and acceleration amplitudes of 25 Hz/4.2 g_rms_ on feet actuators and 15 Hz/0.7 g_rms_ on shoulder actuators were chosen as vibration stimuli. This is because we found that multi-frequency excitation at the distal end of the body stimulated upper extremity muscles to a greater extent than single frequency WBV. There was no slip between the device and body at the interfaces on feet and shoulders. Each of the interfaces had high-density minicell closed cell polyethylene foam lining for better grip and to avoid slip. To reduce the friction between the bed and the participants’ posterior surface, a commercially available smooth plastic sheet was used. LabVIEW (National Instruments, version 2017) was used to simultaneously program and generate frequencies of 25 and 15 Hz. The acceleration amplitudes were controlled from the actuator amplifiers.

### D. ACCELERATION

**FIGURE 3.**
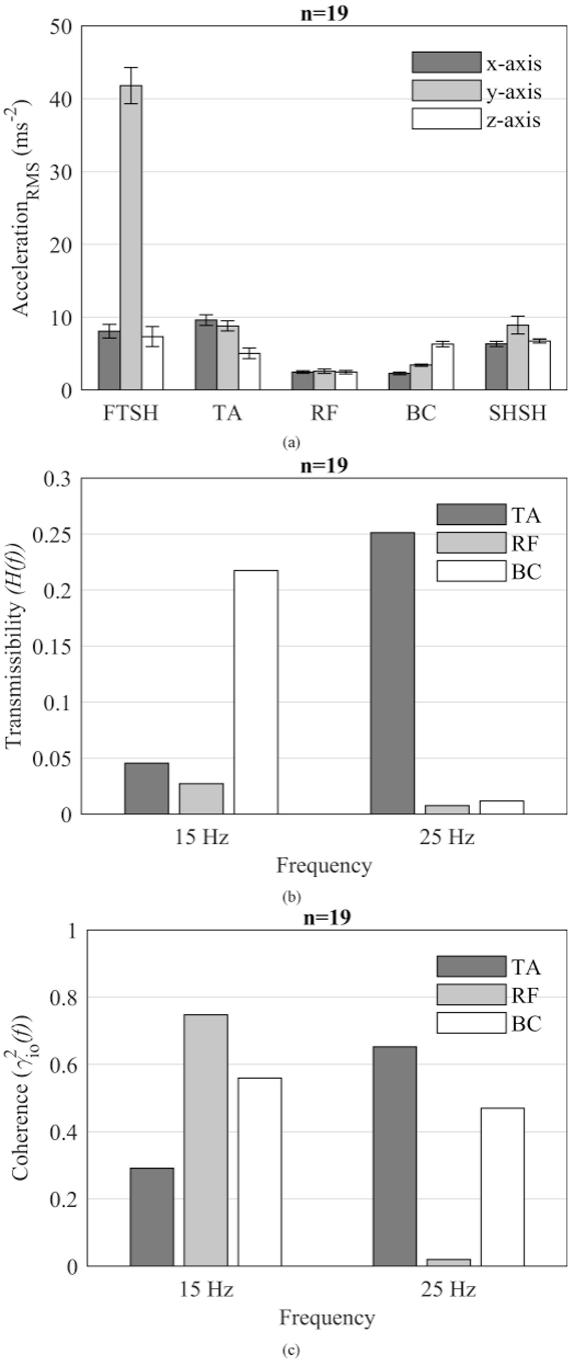
(a) Mean ± SEM of the low pass filtered acceleration on foot shaker (FTSH), tibialis anterior (TA), rectus femoris (RF), brachialis (BC) and shoulder shaker (SHSH). (b) Transmissibility, and (c) Coherence computed using the cross spectral density method from the mean acceleration of all participants in the direction of vibration (refer Fig. 1(b) for the coordinate axes of the accelerometers) at simultaneous excitation frequencies of 15 Hz on shoulders and 25 Hz on feet.

Shimmer3 wireless inertial measurement units (Shimmer, Ireland), each containing a triaxial wide range accelerometer (±16 g), were used to measure accelerations on the actuators and tissue. They were mounted using self-adhesive elastic bandages on the foot actuator, shoulder actuator, tibialis anterior (TA), rectus femoris (RF) and brachialis (BC) muscles. Each sensor has a sampling frequency of 512 Hz, weighs 23.6 grams, 14-bit resolution, and a root mean square noise of 0.6 mg at ±2 g. The data were detrended and then low pass filtered using a 6th order Butterworth filter with a cut-off frequency of 35 Hz. Intra-subject mean root mean square values for 4-second segments for each individual were calculated, and then the inter-subject mean RMS was computed. Using the cross-spectral density method, the transmissibility *H*(*f*) was defined as [48]

$$H(f)=\frac{G_{io}(f)}{G_{ii}(f)}\;,$$

where *G*_*io*_*(f)* is the cross-spectral density between the acceleration on the actuators (input) and the tissue acceleration (output), *G*_*ii*_*(f)* is the power spectral density of the actuator acceleration (input). The transmissibility was interpreted assuming linear elastic behavior of the tissue so that the amplitude of the input did not have to be intentionally varied. The coherence was also calculated to examine the relationship between the output and input signals. The signal coherence was defined as [48]

$$\gamma_{io}^{2}(f)=\frac{|G_{io}(f){|}^{2}}{G_{ii}(f)\;G_{oo}(f)}\;,$$

where *G*_*oo*_*(f)* is the power spectral density of the tissue acceleration. The spectral densities were calculated using the mean acceleration values with Welch’s power spectral density estimate at a resolution of 0.25 Hz (Fig. 3).

### E. MUSCLE ACTIVATION

A BTS FREEEMG 1000 (BTS S.p.A., Italy) system containing wireless EMG probes (13 grams, CMRR >110db at 50-60 Hz, input impedance 100 MΩ) was used to measure muscle activity from eight anatomical sites on the body. The probes were directly attached to bipolar Ag/AgCl pre-gelled disposable snap electrodes (Covidien Kendall disposable surface electrodes H124SG 30 mm ×24 mm, Covidien, USA) and affixed using self-adhesive elastic bandage. The anatomical sites were prepared using an abrasive skin gel (Nuprep, Weaver and Company, USA). The probes were positioned on the muscle bellies of the soleus (SO), tibialis anterior (TA), gastrocnemius lateralis (GL), vastus medialis (VM), vastus lateralis (VL), rectus femoris (RF), semitendinosus (ST) and deltoideus medius (DM). The electrodes were aligned with the muscle axis and placed on the muscle with an inter electrode distance of 20 mm in accordance with the SENIAM (surface electromyography for the non-invasive assessment of muscles) recommendations [49]. Baseline EMG data were recorded prior to commencement of vibration, and a 1 second segment was extracted for post processing. For computing muscle activation during vibration, a 10 second EMG segment was extracted after 1 minute of start of the vibration. The extracted signals were filtered using an 8th order Butterworth digital band-pass filter from 10-400 Hz followed by zero-phase digital filtering to preserve the waveform. A linear interpolation method [50] which filters out only the artifact spikes from the signal in the Welch’s power spectral density was implemented [7], [14], [50]. The spikes corresponding to excitation frequencies of 15 Hz, 25 Hz, 60 Hz (line frequency) and their harmonics up to 400 Hz were filtered out. A uniform spike width of ± 2 Hz around 15 Hz, 25 Hz, 60 Hz and their harmonics were replaced by interpolated straight line. Similar filtering procedures were carried out for EMG signals recorded during MVIC tests and baseline recording. The root-mean square values of the EMG signals of vibration and MVIC were directly calculated from the Welch’s power spectral density. Normalization to MVIC was carried out using the formula, (Vibration EMG_RMS_)/(MVIC EMG_RMS_) × 100. Bias was calculated using the formula (Filtered EMG_RMS_ at baseline)/(Unfiltered EMG_RMS_ at baseline), and the bias-corrected EMG during vibration was computed using (Vibration EMG_RMS_ /Bias) [50].

### F. INDIRECT CALORIMETRY

A COSMED K5 wearable metabolic system (COSMED srl, Italy) was used to measure oxygen uptake (VO_2_), carbon dioxide production (VCO_2_), energy expenditure (EE), minute ventilation (VE) and tidal volume (VT). Testing was conducted in a mixing chamber mode which uses the measuring principle of proportional sampling and collection of exhaled gas via a miniaturized mixing chamber. Based on the manufacturer’s guidelines, the calibration of the device was carried out at recommended intervals. The turbine flowmeter was calibrated before each test using the manufacturer’s supplied 3-liter calibration syringe. The zero of the CO_2_ analyzer and detection of environmental air composition was performed using the manufacturer’s supplied scrubber. The oxygen sensors were calibrated using the manufacturer’s supplied calibration gas cylinder with recommended concentration of 16% oxygen, 5% carbon dioxide, and balance nitrogen. Data were sampled at 0.1 Hz (i.e., every 10 seconds) and updated using a 30 second moving average. The calorimetry data were recorded in supine position both at baseline and during vibration. For the baseline data, a mean of 3 minutes (18 data points) of the segment preceding vibration was computed. For the data collected during vibration, a moving average peak analysis for every 3 minutes for 10 minutes of VO_2_, VCO_2_, EE, VE, and VT data was carried out. The maximum value of the moving average was selected as the mean value of VO_2_. This methodology of segment extraction precluded the possibility of picking up short transient changes in metabolic data and helped in ensuring selection of steady set of values of metabolic variables which estimated the true response of the participant.

### G. REGIONAL HEMOGLOBIN OXYGEN SATURATION

A Nonin SenSmart Model X-100 Universal Oximetry System (Nonin Medical, Inc., USA) was used to measure rSO_2_ using near-infrared spectroscopy. Oxygenation sensors were placed on three anatomical sites i.e., the gastrocnemius lateralis (GL), rectus femoris (RF) and biceps brachii (BB). The rSO_2_ data were recorded at a sampling frequency of 0.25 Hz (i.e., once every 4 seconds). The data were filtered by the manufacturer’s signal processing algorithms to reduce interference from external sources and motion artifacts. The mean value of rSO_2_ for 1 minute (15 data points) preceding vibration was computed as the baseline value. For the data collected during vibration, a moving average peak analysis for every 1 minute for 10 minutes of rSO_2_ data was carried out. The maximum value of the moving average was selected as the mean value of vibration. The moving average peak analysis was independently conducted for all three measurements from GL, RF and BB.

### H. DATA PROCESSING AND STATISTICS

Data processing of EMG, rSO_2_ and metabolic data were performed using MATLAB version 9.4 R2019a (MathWorks, MA, USA). All values are expressed as mean ± SEM (standard error of the mean) unless otherwise stated. Statistical analyses were performed using IBM SPSS Statistics version 25 (SPSS Inc., Chicago, IL). A one-way repeated measures analysis of variance (ANOVA) with condition (baseline and vibration) as a repeated measure was carried out to test the effect of WBV on muscle activation, metabolic parameters, and rSO_2_%. Statistical significance level was set at *p* < 0.05 for all tests.

## III. RESULTS

### A. MUSCLE ACTIVATION

**FIGURE 4.**
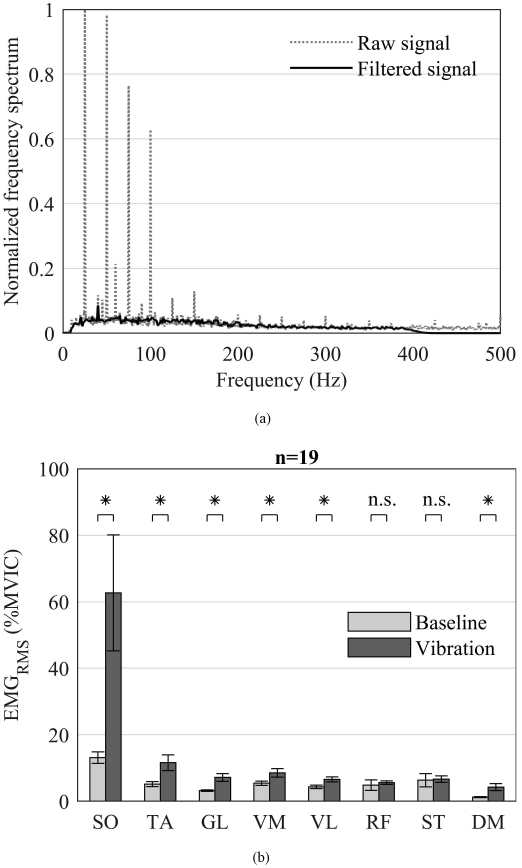
(a) A sample EMG frequency spectrum of the soleus (SO) muscle from a single participant in a single session at multi-frequency synchronous excitation of 15 Hz on shoulders and 25 Hz on feet. The gray dotted line depicts the raw EMG signal and the black solid line depicts the band-pass (8th order Butterworth filter 10-400 Hz), linear interpolated filtered EMG signal. The motion artifacts (sharp spikes) at excitation frequencies and corresponding harmonics up to 400 Hz have been removed using the linear interpolation method. (b) Mean ± SEM of the filtered EMG RMS at baseline and during vibration normalized to the maximum voluntary isometric contraction (MVIC) at excitation frequencies of 15 Hz and 25 Hz. EMG measured from the muscle bellies of soleus (SO), tibialis anterior (TA), gastrocnemius lateralis (GL), vastus medialis (VM), vastus lateralis (VL), rectus femoris (RF), semitendinosus (ST) and deltoideus medius (DM). Asterisks indicate statistical significance at *p* < 0.05 and n.s. indicates not significant.

There was a significant effect of condition on muscle activation [F (8, 11) = 17.17, *p* < 0.0001, Wilk’s *Λ* = 0.074, partial *η*^2^ = 0.926]. Post hoc analysis with Bonferroni correction indicated that the mean activation was significantly higher during WBV for the SO (*p* = 0.011), TA (*p* = 0.012), GL (*p* = 0.003), VM (*p* < 0.001), VL (*p* < 0.0001), DM (*p* = 0.006), but not for RF (*p* = 0.59) and ST (*p* = 0.86) (Fig. 4).

### B. INDIRECT CALORIMETRY

**FIGURE 5.**
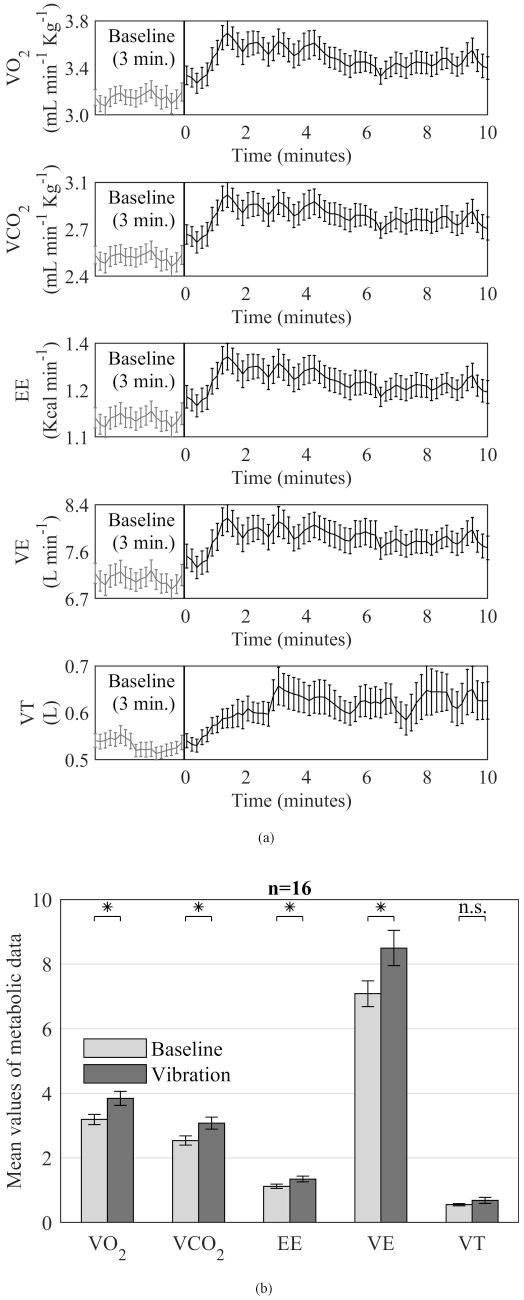
(a) Mean ± SEM of each metabolic data point in time series at baseline and during vibration. (b) Mean ± SEM of oxygen uptake (VO_2_), carbon dioxide production (VCO_2_), energy expenditure (EE), minute ventilation (VE), and tidal volume (VT) recorded at rest and during vibration. Asterisks indicate statistical significance at *p* < 0.05 and n.s. indicates not significant.

There was a significant effect of condition on metabolic data [F (5, 11) = 7.42, *p* = 0.003, Wilk’s *Λ* = 0.229, partial *η*^2^ = 0.771]. Post hoc analysis with Bonferroni correction indicated significant increases in VO_2_, VCO_2_, EE, VE (*p* < 0.0001) but not for VT (*p* = 0.094) during vibration compared with baseline period (Fig. 5).

### C. REGIONAL HEMOGLOBIN OXYGEN SATURATION

**FIGURE 6.**
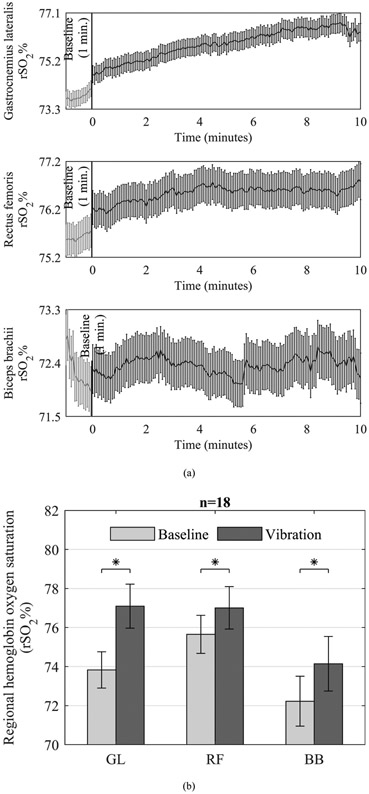
(a) Mean ± SEM of each regional hemoglobin oxygen saturation (rSO_2_%) data point in time series at baseline and during vibration. (b) Comparison of rSO_2_% (mean ± SEM) at rest and during vibration. Asterisks indicate statistical significance at *p* < 0.05.

There was a significant effect of condition on rSO_2_ data [F (3, 15) = 20.93, *p* < 0.0001, Wilk’s *Λ* = 0.193, partial *η*^2^ = 0.807]. Post hoc analysis with Bonferroni correction indicated that the mean rSO_2_ of GL (*p* < 0.0001), RF (*p* < 0.0001) and BB (*p* < 0.001) were significantly higher during vibration compared with baseline period (Fig. 6).

## IV. DISCUSSION

The purpose of this study was to test the effects of simultaneous multi-frequency WBV delivered axially using a therapeutic vibration device on several important physiological markers of physical activity in healthy participants as a pilot study in prelude to a future study on critically ill immobilized subjects. Results indicate that WBV using the therapeutic vibration device induced modest but significant increases in muscle activation, regional muscle oxygenation, and metabolic parameters. These increases were similar to those observed during mild physical activity, suggesting that WBV might serve as a suitable approach in assisting to combat muscle weakness and associated dysfunctions of PICS due to prolonged immobilization.

### A. MUSCLE ACTIVATION DURING WBV

Several studies have evaluated the effects of WBV on muscle activation and have shown that WBV increases EMG activation of both the upper- and lower-extremity muscles [7]-[19]. However, such WBV protocols are often carried out in standing position or with added loads through active contraction of the individual’s muscles, which may not be applicable to critically ill patients who are often sedated and unconscious. Moreover, WBV while standing or exercising is often accompanied by posture dependent voluntary contractions, which may confound the results by masking the actual muscle activity induced by the vibration. To our knowledge, this is the first study to characterize the effects of WBV on muscle activation using a novel therapeutic vibration device that was specifically designed for the treatment of immobilized critically ill patients in the intensive care unit. We found that WBV using the therapeutic vibration device significantly increased the muscle activation of upper and several lower-extremity muscles. We note that the observed increase in muscle activation was much larger in the distal leg muscles than in the proximal muscles, which is a finding that is consistent with previous studies [7], [12]. A key reason for this observation is that the acceleration amplitude on body segments was substantially dampened as the distance from the source of vibration increased (Fig. 3). Further, it is possible that the preloading of the muscles using the restraint mechanism may not have been adequate for the proximal muscles.

It is important to note that the EMG values were expressed as a percentage of MVIC values. Therefore, a formal statistical comparison between the EMG values recorded during MVIC and during baseline or vibration was not possible. While the increase in EMG due to WBV did not reach MVIC values (as shown in Fig. 4), it is unrealistic to expect such a drastic increase in EMG activation via WBV without any added exercise. We did not incorporate any additional exercise and sought to study the isolated effects of WBV because patients who are in a critical condition are often sedated and paralyzed, and hence, will not have the capability to perform any volitional exercise. Moreover, they will not respond to other promising interventions such as neuromuscular electrical stimulation. Thus, WBV is the only alternative for providing mechanical stimulus to these patients, and this study provides the initial foundation for future studies that are planned on critically-ill patients. Further, a small change in muscle activation in multiple muscles could be strongly beneficial to critically ill patients, albeit, this remains to be tested. Thus, future research should focus on optimizing the vibration frequency and loading of the muscles to maximize the clinical potential of the therapeutic vibration device.

### B. INDIRECT CALORIMETRY DURING WBV

WBV, when combined with exercise, is known to augment VO_2_ and energy expenditure [28]-[31]. However, the ability of WBV to improve metabolic consumption while lying relaxed has remained somewhat unclear. Here, we show for the first time that meaningful improvements in metabolic consumption (22.3%) can be induced by WBV in recumbent position using the therapeutic vibration device. Previous studies have suggested that an increase in metabolic rate during WBV is due to increased muscle activation [20]-[22], [32] and tissue oxygenation [22], [32]. Additionally, the application of external loads during WBV has been shown to enhance metabolic rate [21], [25]-[27]. The results of the current study using preloading are in general, consistent with these previous reports demonstrating an increase in VO_2_, VCO_2_, energy expenditure, and minute ventilation, which can be interpreted as a response to increased musculature metabolic demand. Apart from the increase in metabolic demand due to WBV, changes in skin/body temperature could also explain the observed increase in VO_2_ [51]. Unfortunately, we did not evaluate changes in skin/body temperature due to vibration in this study. While there is no reason to believe that skin/body temperature could have changed due to extraneous factors, as the room temperature was maintained constant and the subjects were resting in recumbent position throughout the experiment, WBV in itself could likely have elevated skin/body temperature [47], [52], [53]. Future research should explore the associations and interactions between muscle activation, skin/body temperature, and VO_2_ to determine the causal relationship between these variables and to establish the mechanistic underpinnings of increased VO_2_ due to WBV.

### C. REGIONAL HEMOGLOBIN OXYGEN SATURATION DURING WBV

Several studies have investigated the acute effects of vibration on peripheral blood flow, venous return, muscle oxygenation, and total hemoglobin and have reported conflicting or contrasting conclusions [22], [32]-[40], [54], [55]. Our results indicate that WBV increases regional hemoglobin oxygen saturation of the upper- and lower-extremity muscles, which is consistent with several previous studies [22], [33]-[35], [38], [40]. While the exact mechanism for the increased oxygen saturation is not clear, it has been previously hypothesized that vibration applied to the bottom of the foot creates a “skeletal muscle pumping” action resulting in displacement of blood, peripheral lymphatic transport, and an increased arteriovenous pressure gradient, which consequently elevates regional oxygen saturation in the tissues [22], [33], [34], [36]-[38]. Additionally, muscle activation due to tonic vibration reflex may trigger metabolic demand and vasodilation, thus leading to enhancement in blood oxygenation and perfusion [32], [36]. Hence, we believe that both mechanisms (i.e., skeletal muscle pumping and increased muscle activation) could have contributed to the observed increase in regional oxygen saturation.

### D. STUDY LIMITATIONS

There are some potential limitations to this study. This study did not examine the isolated effects of shoulder (15 Hz) and foot (25 Hz) vibration because our primary question was related to whole body vibration. Moreover, our pilot testing indicated that some of the measured parameters did not return to baseline even after 10 minutes of completion of the vibration, which would necessitate a large amount of washout period. However, because of our experimental study design, it is not clear if the effects of shoulder and foot vibration differ from each other and if the effect of the 15 Hz is masked, amplified, or negated by the 25 Hz frequency. Further research is needed to study the isolated effects of shoulder and foot vibration on various physiological parameters as this will help in optimizing vibration dosage for upper and lower extremity muscles. Another limitation of this study is that it may be difficult to fully extrapolate the results of the present study to patients at risk for PICS from prolonged immobilization. The participants in this study were medically healthy and led relatively active lifestyles. It is not clear if critically ill and injured patients who are immobilized would realize physiologic responses similar to or greater than those of this healthy cohort. Further, it is not known from this study whether the degree of muscle activation changes observed with WBV would be sufficient to act as a significant mitigator of atrophy. We also do not know what effect it may have at the center of the body, as we did not measure any parameters (e.g., EMG or rSO_2_%) at this region. As can be seen in Fig. 3, the acceleration amplitude substantially dampens in proximal muscles, which is due to the nonlinear behavior of the human body [7], [12] and inadequate preloading of the trunk/center of the body muscles. Based on this observation, we speculate that the transmission to the center of the body is relatively low compared to the extremities, which could result in low muscle activation and other physiologic responses. Future studies should evaluate the effects of WBV at the trunk region as it could have meaningful therapeutic implications for lung health in critically ill patients (e.g., sputum clearance). Additionally, measurement of myoglobin would have provided insight into the muscle metabolism due to muscle activation. Unfortunately, this study did not measure myoglobin due to the Nonin SenSmart X-100 NIRS device limitations. However, in our future studies we will measure myoglobin to gain insight into muscle metabolism due to therapeutic vibration device. Finally, this study did not address dosing in terms of frequency or duration of vibration on various physiologic parameters. Thus, future studies are needed to address the issue of dosage and also to examine both acute and long-term effects of WBV using the therapeutic vibration device in critically ill and injured patients to determine its potential to counteract PICS due to prolonged immobilization.

## V. CONCLUSION

This study tested the effects of simultaneous multi-frequency axial WBV using inertial actuators on physiological parameters in healthy individuals. The results of the study indicate that WBV induces significant changes in muscle activation, metabolic parameters of oxygen consumption, and tissue oxygenation that are consistent with mild physical activity. Additional studies on individuals who are immobilized due to critical illness are needed to understand if WBV using the therapeutic vibration device may act as a sufficient countermeasure to prevent PICS associated atrophy and its complications.

## Figures and Tables

**TABLE 1.** Abbreviation list.

Abbreviation	Description
WBV	Whole body vibration
TVD	Therapeutic vibration device
ICU	Intensive care unit
PICS	Post-intensive care syndrome
MVIC	Maximal voluntary isometric contraction
EMG	Electromyography
rSO_2_%	Regional hemoglobin oxygen saturation
SO	Soleus
TA	Tibialis anterior
GL	Gastrocnemius lateralis
VM	Vastus medialis
VL	Vastus lateralis
DM	Deltoideus medius
RF	Rectus femoris
ST	Semitendinosus
BB	Biceps brachii
BC	Brachialis
VO_2_	Oxygen uptake
VCO_2_	Carbon dioxide production
EE	Energy expenditure
VE	Minute ventilation
VT	Tidal volume
FTSH	Foot shaker
SHSH	Shoulder shaker
SENIAM	Surface electromyography for the non-invasive assessment of muscles
EMG_RMS_%	RMS of EMG values expressed as a percentage of MVIC
SEM	Standard error of the mean
